# Signal processing of heart signals for the quantification of non-deterministic events

**DOI:** 10.1186/1475-925X-10-10

**Published:** 2011-01-26

**Authors:** Véronique Millette, Natalie Baddour

**Affiliations:** 1 Department of Mechanical Engineering, 161 Louis Pasteur, University of Ottawa, K1N 6N5, Ottawa, Ontario, Canada

## Abstract

**Background:**

Heart signals represent an important way to evaluate cardiovascular function and often what is desired is to quantify the level of some signal of interest against the louder backdrop of the beating of the heart itself. An example of this type of application is the quantification of cavitation in mechanical heart valve patients.

**Methods:**

An algorithm is presented for the quantification of high-frequency, non-deterministic events such as cavitation from recorded signals. A closed-form mathematical analysis of the algorithm investigates its capabilities. The algorithm is implemented on real heart signals to investigate usability and implementation issues. Improvements are suggested to the base algorithm including aligning heart sounds, and the implementation of the Short-Time Fourier Transform to study the time evolution of the energy in the signal.

**Results:**

The improvements result in better heart beat alignment and better detection and measurement of the random events in the heart signals, so that they may provide a method to quantify nondeterministic events in heart signals. The use of the Short-Time Fourier Transform allows the examination of the random events in both time and frequency allowing for further investigation and interpretation of the signal.

**Conclusions:**

The presented algorithm does allow for the quantification of nondeterministic events but proper care in signal acquisition and processing must be taken to obtain meaningful results.

## 1. Background

Listening to the heart is perhaps the most important, basic, and effective clinical technique for evaluating a patient's cardiovascular function. A skilled practitioner can quickly evaluate common complaints that may be quite serious. In recent years, there has been an interest in applying signal processing techniques to aid in the detection, analysis and quantification of various aspects of interest in heart signals. Signal processing can be used to automate the measurement of various signal characteristics, reducing subjectivity and increasing reliability. Another purpose is to filter out undesired signal components with either technical or physiological origin so that analysis of the relevant portion of the signal is facilitated. For example, some analyses have focused on attempting to identify valve abnormality [1,2], heart rate variability [3-5], to detect heart pathologies [6-10], to detect murmurs [11] and to detect and quantify cavitation in mechanical heart valve patients [12-20].As can be seen from this brief list, signal processing algorithms of heart signals usually have the goal of isolating a random or non-deterministic component (such as a murmur or cavitation) from the comparatively loud backdrop of the beating of the heart itself.

As a specific example of this type of application, the issue of cavitation in mechanical heart valve patients was first recognized when damaged mechanical heart valves were observed. Cavitation bubble implosion can cause mechanical damage to the valve structure and blood elements when it occurs near the surface of the mechanical heart valve. Cavitation in a fluid can be detected acoustically or visually. But since blood is not a transparent fluid, the cavitation near a mechanical heart valve has to be detected acoustically for in vivo studies. The acoustic evidence of cavitation is defined by the high-frequency pressure fluctuations (HFPFs) associated with transient bubble collapse [16]. These HFPFs can be detected acoustically with the use of a high sensitivity hydrophone by applying it on the patient's chest since a hydrophone can record high frequency sounds [13]. It is thought that the HFPFs may provide information regarding the intensity and duration of cavitation [16].

A common theme to all these signal processing applications for heart signals is that the sound measured includes a component coming from the heart itself as well as a signal of interest, (cavitation or otherwise). To obtain the signal of interest, the repetitive heart-beat component has to be removed from the signal [16]. For the quantification of cavitation, a few methods were proposed to remove this component from the signal and to quantify the level of cavitation by Garrison *et al. *[14], Johansen *et al. *[18,21], Sohn *et al. *[22]and Herbertson *et al. *[15].

However, none of these proposed signal processing methods have been investigated for the issues of usability and robustness with real heart-beat signals. In this paper, we undertake an analytical and experimental investigation into signal processing of real heart-beat signals acquired in-vivo for the purpose of quantifying high-frequency signals of interest (such as cavitation) against the backdrop of the heart-beat itself. The focus is on usability with real heart-beat signals, which are by nature noisy and not perfectly periodic.

To accomplish these goals, we propose and validate an algorithm for the quantification of non-deterministic (non-repetitive) events in a heart signal. The main motivation and context of this algorithm stems from cavitation detection and quantification [18,21], however the authors suggest that this algorithm could potentially be useful for a larger class of applications. The two algorithms by Johansen *et al. *from the literature are the basis for the algorithm. These algorithms were chosen as they presented the most potentially effective algorithm in the literature to date that had actually been implemented on biological signals acquired in vivo.

It is emphasized that the goal of the paper is to investigate usability and robustness of the chosen signal processing algorithms, in essence to validate a tool that can be used to establish appropriate levels of non-deterministic energy of a quantity of interest embedded in the background of heart-beat. The actual levels of non-deterministic energy of a quantity of interest, such as cavitation, that are considered normal in a given population would require a separate study, once the appropriate signal processing tools have been chosen for that investigation. The aim is thus to present at tool that can be used by other researchers to determine what those appropriate levels might be.

## 2. Methods

The algorithm to be presented is based on ideas proposed by Johansen for the detection of cavitation in mechanical heart valve patients [18,21]. In what follows, we set this algorithm on a firm mathematical foundation via a rigorous analysis and we show that theoretically, it should quantify the quantities we seek to capture. The algorithm is then implemented on real heart-beat acoustic signals obtained via a stethoscope or high frequency hydrophone with healthy subjects. We seek to evaluate the algorithm with real-life signals and their inherent difficulties, rather than 'fake' test signals. In particular, slight changes in the length of each heart-beat and typical levels of noise could pose difficulties. Low-pass filtering for noise removal is not performed as it would also likely remove the signal-of-interest (usually a high frequency component). Thus the algorithm will have to be able to deal with higher levels of noise than might be desirable.

By using signals from healthy subjects, no cavitation or abnormalities are expected to occur in the signal. Since no cavitation or other non-deterministic features of interest are expected in the signal then a quantitative measure of the "goodness" of the algorithm is lower calculated levels of "potential features of interest". A change to the algorithm that lowers the levels of non-deterministic markers is considered to be improving the algorithm. This is an important point to make as this allows us to work with real heart signals.

For implementation of the algorithm, both stethoscope and hydrophone signals were used. Some of the stethoscope signals that were used in the algorithms were pre-recorded stethoscope test signals that were obtained from a CD provided with the Littmann stethoscope. Other signals that were used in the algorithms are in-vivo stethoscope signals recorded in our lab on ourselves, representing healthy subjects. Those recordings were made with the same electronic stethoscope, sound card, and laptop as described below. The acquisition of these signals was in compliance with the University of Ottawa Ethics Guidelines.

For further analysis with other types of signals, in-vitro recordings were also obtained using a left-heart simulator in a laboratory at the University of Ottawa Heart Institute [23]. The left-heart simulator is a mechanical system that simulates the activity in the left portion of the heart and is designed for in vitro testing of bioprosthetic and mechanical heart valves. The left heart simulator used in this experiment was purchased from ViVitro Labs Inc. (Victoria, Canada). Designed to be physiologically realistic, it can assess the function of heart valves and other devices under simulated cardiac conditions. The simulator consists of a Superpump system, a viscoelastic impedance adapter, a left heart model, flow and pressure measuring systems, a waveform generator, and a PC data acquisition system. The valves used in this experiment were a bioprosthetic valve of type Magna from Edwards Lifescience (Irvine, California, USA), and a St. Jude Medical bileaflet mechanical heart valve. The bioprosthetic valve was an aortic trileaflet bovine pericardial valve with a commercial denomination of 27 mm. The mechanical heart valve had pyrolytic carbon occluders. Its interior diameter was 18 mm and its exterior diameter including the cuff was 28 mm. Its commercial denomination was 23 mm. Cavitation was not visually observed with the use of either valve. Full details are given in [24].

**Figure 1:**
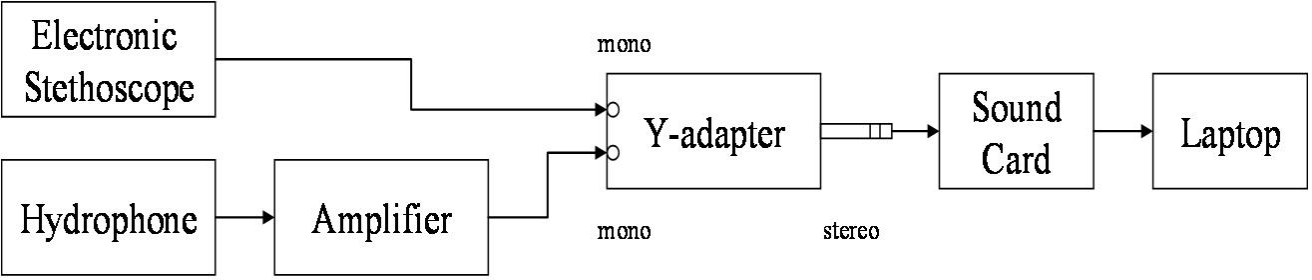
**Flow chart of the experimental setup for the data gathering**.

The high-frequency pressure fluctuations were also measured using a miniature hydrophone. The hydrophone used is the 8103 miniature hydrophone by Brüel & Kjaer, Naerum, Denmark. Its frequency range is 0.1 Hz to 180 kHz. The hydrophone was connected to the Nexus Conditioning Amplifier by Brüel & Kjaer. To help segment the hydrophone signal, an electronic stethoscope was positioned beside the hydrophone, and the stethoscope recording was done simultaneously with the hydrophone recording. The electronic stethoscope used was the Welch Allyn Elite Electronic Stethoscope, New York, USA. Its frequency range is 20 Hz to 20 kHz. The stethoscope and hydrophone were connected to a Y-adapter, which was connected to the sound card. The Y-adapter used was a dual mono jack to stereo plug adapter. The sound card used in this research was the Creative Sound Blaster Audigy 2 ZS Notebook sound card. It was capable of recording with a sampling rate of up to 96 kHz. To maintain a manageable data set, a sampling rate of 44.1 kHz was used for healthy subjects since no cavitation was expected. The sampling rate could be changed to 96 kHz when cavitation was expected. Finally, the sound card was plugged into the laptop (ASUS A3E) where the data were stored. The data was recorded with software called Creative Smart Recorder which came with the sound card. Figure 1 illustrates the equipment used and the experimental setup.

### 2.1. The Algorithm

#### 2.1.1. Rationale

It has been suggested in [18] and [21] by Johansen *et al. *that the cavitation near mechanical heart valves can be quantified by separating the acoustic pressure signal into deterministic and non-deterministic components. The deterministic component represents the valve closing sound assumed deterministic since valve closure is cyclic. The non-deterministic component is the information of interest. This was assumed to originate from cavitation but may stem from other 'signals of interest' such as heart-murmurs, thus widening the applicability of this algorithm. The non-deterministic component of the signal also contains unwanted random noise from various sources such as the detection equipment used.

Based on this, the algorithm follows on the assumption that the information of interest is quantified by the level of non-deterministic energy, defined as the difference between the deterministic energy and the total energy:

(1) $$E_{\text{non-det}}=E_{\text{total}}-E_{\text{det}}$$

Here *E*_non-det _represents the non-deterministic signal energy which is the energy of interest, attributed to the random, non-deterministic events of interest. *E*_total _represents the total signal energy containing all energies and *E*_det _represents the deterministic signal energy of the repeating components of the signal that we seek to eliminate, the heart beat itself. How these energies are defined mathematically will be presented subsequently. Since the quantity of interest is a often a high-frequency component [16,18,21], no low-pass filtering is applied to the original signal.

#### 2.1.2. The algorithm

The first step in the algorithm is to segment the total signal into individual heartbeats and then line up all the heart beats in time. Then, each heart beat is truncated so that all beats have the same length since vectors of different lengths cannot be averaged. The end part of the beats is truncated since no important information is located there. The truncated heart beats are then superimposed one over the other and ensemble averaged to obtain an average heart beat signal. This theoretically eliminates unwanted noise as well as reduces the signal parts that do not repeat from beat to beat.

The next step in this algorithm is then to find the deterministic energy. This is defined as

(2) $$E_{\text{det}}=\frac{1}{N}\sum_{n=1}^{N}{\left|F\left(p_{ea}[n]\right)\right|}^{2}$$

where *N *is the number of samples in each heart beat, *p*_*ea*_[*n*] is the ensemble average of the heart beats, and *F *is the Fourier Transform. The ensemble average is the average heartbeat calculated according to

(3) $$p_{ea}[n]=\frac{1}{HB}\sum_{m=1}^{HB}p_{xy}[n,m]$$

where *HB *is the number of heart beats measured, and *p*_*xy*_[*n*,*m*] represents the *n*^th ^sample of the *m*^th ^heart beat in the total signal.

The third step in this algorithm consists of determining the total energy. The energy is determined for each segmented and truncated heart beat and then these energies are averaged, which represents the total energy. Mathematically,

(4) $$E_{\text{total}}=\frac{1}{HB}\sum_{i=1}^{HB}\left(\frac{1}{N}\sum_{n=1}^{N}{\left|F\left(p_{xy}[n,i]\right)\right|}^{2}\right)$$

where |*F*(*p*_*xy*_[*n*,*i*])|^2^is the amplitude spectrum squared of the *i*^th ^heart beat.

**Figure 2:**
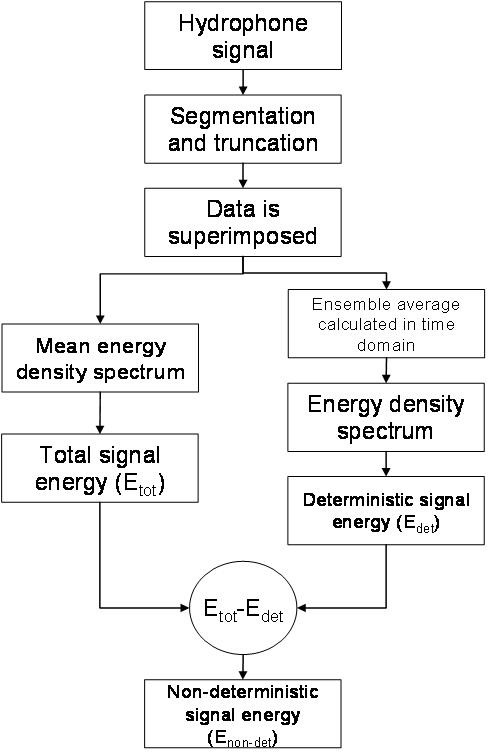
**Block diagram summarizing the algorithm**.

The final step consists of subtracting the deterministic energy from the total energy to obtain the non-deterministic energy. Figure 2 is a block diagram summarizing the algorithm.

#### 2.1.3. Segmentation algorithm

The first step of the algorithm is to segment the signal into individual heartbeats. The segmentation problem is a topic of research in itself and we do not attempt a literature review of the subject here. Many algorithms have been proposed in this area, for example [25-36] and any suitable segmentation algorithm can be chosen for this step. The segmentation algorithm used in this paper was the method developed in [37]. This algorithm was used instead the cross-correlation method proposed by Johansen since the results obtained by Johansen [18] could not be reproduced.

The Courtemanche segmentation algorithm used herein proposed the use of an adaptive threshold wavelet transform filtering technique used with Shannon energy, physiological factors and heart rate approximation to properly identify the first heart sounds (S1) and segment the heart signal. However, this method can still present some errors when faced with complex signals. Therefore, the addition of a Mel-Scaled Wavelet Transform (MSWT) validation step was proposed. The MSWT is a modified Mel-Frequency Cepstral Coefficient (MFCC) algorithm with the Discrete Wavelet Transform (DWT), and it was used to reduce the impact of noise on the coefficients. The preliminary results obtained in the paper indicated that the MSWT is less prone to noise than the MFCC and can distinguish S1 sounds from others when faced with complex signals [37].

The segmentation points obtained using the Courtemanche segmentation algorithm [37] were compared with those obtained using a different segmentation algorithm as suggested in [38]. The stethoscope signals were independently segmented by the first author of [38], and it was found that the segmentation points obtained were very similar to the ones obtained using the Courtemanche algorithm of [37]. This served to verify the calculation of the segmentation points.

### 2.2. Mathematical analysis of the algorithm

To ensure that the algorithm should indeed quantify the non-deterministic signals of interest, a rigorous mathematical analysis is presented. To simplify the mathematical analysis, Parseval's relation is used so that the energy of a signal can be equivalently found from the time or frequency domain.

#### 2.2.1. Calculations with a continuous signal x(t)

##### Deterministic energy

Analytically, the segmentation and truncation of the original signal is accomplished by multiplying the continuous signal *x*(*t*) by a rectangular window *w*_*n*_(*t*) at time *t*_*n*_and having a length of one truncated heart beat *T. *The truncated heart beats are then shifted, superimposed and then averaged to obtain an average heart beat signal given by

(5) $$z(t)=\frac{1}{HB}\sum_{n=1}^{HB}y_{n}(t)$$

where each time-shifted heart beat *y*_*n*_(*t*) = *y*(*t *+ *t*_*n*_) is found by shifting each heart beat back to time zero, after multiplying the original signal with the window *y*(*t*) = *x*(*t*)·*w*_*n*_(*t*) to obtain each beat. The deterministic energy is the energy of the ensemble-averaged heart beat:

(6) $$E_{\det}=\int_{-\infty }^{+\infty }{\left|z(t)\right|}^{2}dt$$

These steps can be illustrated with *n *heartbeats but for brevity, the calculation is illustrated with three heart beats. For three heart beats, we have that

(7) $$\begin{array}{l} z(t)=\frac{1}{3}\sum_{n=1}^{3}y_{n}(t)\\ \;\;\;\;\;\;=\frac{1}{3}y_{1}(t)+\frac{1}{3}y_{2}(t)+\frac{1}{3}y_{3}(t) \end{array}$$

Hence the deterministic energy is given by

(8) $$\begin{array}{l} E_{det}=\int_{-\infty }^{+\infty }{\left|z(t)\right|}^{2}dt\\ \;\;=\;\frac{1}{9}\int_{-\infty }^{+\infty }y_{1}^{2}(t)dt+\frac{1}{9}\int_{-\infty }^{+\infty }y_{2}^{2}(t)dt+\frac{1}{9}\int_{-\infty }^{+\infty }y_{3}^{2}(t)dt\\ \;\;+\underset{\text{Cross terms}}{\underset{︸}{\frac{2}{9}\int_{-\infty }^{+\infty }y_{1}(t)y_{2}(t)dt+\frac{2}{9}\int_{-\infty }^{+\infty }y_{1}(t)y_{3}(t)dt}}\\ \;\;+\underset{\text{Cross term}}{\underset{︸}{\frac{2}{9}\int_{-\infty }^{+\infty }y_{2}(t)y_{3}(t)dt}} \end{array}$$

If *y*_1_(*t*) = *y*_2_(*t*) = *y*_3_(*t*), implying that the three heart beats are identical and perfectly superimposed, then

(9) $$E_{\det}=\int_{-\infty }^{+\infty }y_{1}^{2}(t)dt$$

This pattern is the same for *n *heartbeats. As observed in (8) compared to (9), if the heart beats are not perfectly superimposed, cross-terms appear in the deterministic energy result, which impacts both the deterministic and thus non-deterministic energy results. This demonstrates the importance of the correct segmentation of the original signal. Imperfect segmentation leads to additional terms in the deterministic energy calculation, and the source of these terms is entirely from the imperfect segmentation and not noise or cavitation. This would, in turn, affect the non-deterministic energy not because of any true additional non-deterministic energy in the signal but rather through imperfect processing of the signal.

##### Total energy

The total energy is defined as the average of individual heart beat energies:

(10) $$E_{total}=\frac{1}{HB}\sum_{n=1}^{HB}\int_{-\infty }^{+\infty }{\left|y_{n}(t)\right|}^{2}dt\;$$

To demonstrate for three heart beats, the total energy is given by

(11) $$\begin{array}{l} E_{total}=\frac{1}{3}\sum_{n=1}^{3}\int_{-\infty }^{+\infty }{\left|y_{n}(t)\right|}^{2}dt\\ \;=\frac{1}{3}\left(\int_{-\infty }^{+\infty }y_{1}^{2}(t)dt+\int_{-\infty }^{+\infty }y_{2}^{2}(t)dt+\int_{-\infty }^{+\infty }y_{3}^{2}(t)dt\right) \end{array}$$

Then if the heartbeats are identical and perfectly superimposed so that *y*_1_(*t*) = *y*_2_(*t*) = *y*_3_(*t*), it follows that

(12) $$E_{total}=\int_{-\infty }^{+\infty }y_{1}^{2}(t)dt$$

Similar results are obtained with *n *beats.

##### Non-deterministic energy

The non-deterministic energy is obtained by subtracting the deterministic energy from the total energy. Continuing our example for three heart beats, the non-deterministic energy is

(13) $$\begin{array}{l} E_{non-\det}=E_{total}-E_{\det}\\ \;\;\;\;=\;\;\frac{1}{3^{2}}\int_{-\infty }^{+\infty }{\left(y_{1}(t)-y_{2}(t)\right)}^{2}dt\\ \;\;\;\;+\;\frac{1}{3^{2}}\int_{-\infty }^{+\infty }{\left(y_{1}(t)-y_{3}(t)\right)}^{2}dt\\ \;\;\;\;+\;\frac{1}{3^{2}}\int_{-\infty }^{+\infty }{\left(y_{2}(t)-y_{3}(t)\right)}^{2}dt \end{array}$$

For beats that are identical, then *E*_*non*__-det _= 0. This clearly demonstrates that the proposed algorithm, so far in theory, works perfectly. Namely, if a signal consists of heart beats that repeat perfectly and can be segmented perfectly, then the non-deterministic energy should be zero.

Suppose that an additional component is added to the signal so that the beats are not identical but are 'similar'. This is done by considering one beat to be fundamentally the same as another but with the addition of an extra signal, which may be cavitation or noise, etc. This is modelled mathematically as

(14) $$\begin{array}{l} \;y_{2}(t)=y_{1}(t)+\eta_{1}(t)\\ \;y_{3}(t)=y_{1}(t)+\eta_{2}(t) \end{array}$$

where *η*_*i*_(*t*)represent cavitation, noise or some other signal of interest. Then the non-deterministic energy calculation gives

(15) $$\begin{array}{l} E_{non-\det}=\underset{\text{Represents cavitation/noise}}{\underset{︸}{\frac{2}{9}\int_{-\infty }^{+\infty }\eta_{1}^{2}(t)dt}}\\ \;\;+\underset{\text{Represents cavitation/noise}}{\underset{︸}{\frac{2}{9}\int_{-\infty }^{+\infty }\eta_{2}^{2}(t)dt-\frac{2}{9}\int_{-\infty }^{+\infty }\eta_{1}(t)\eta_{2}(t)dt}} \end{array}$$

The previous equation again demonstrates analytically that the proposed algorithm is a quantitative measure that contains *only *the non-repeating elements of a signal since the repeating portions of the signal do not appear in the expression for non-deterministic energy. Therefore, the theory behind the algorithm is sound.

#### 2.2.2. Effect of beat misalignment

In the case when the heart beats are not perfectly superimposed, the non-deterministic energy result is affected. The impact is demonstrated analytically with two heart beats. We start with the non-deterministic energy but now consider the case where *ε *represents the factor by which the second heart beat is identical to the first but misaligned with respect to it so that *y*_2_(*t*) = *y*_1_(*t + ε*). This gives

(16) $$\begin{array}{l} E_{non-\det}=\frac{1}{4}\int_{-\infty }^{+\infty }y_{1}^{2}(t)dt\\ \;+\frac{1}{4}\int_{-\infty }^{+\infty }y_{1}^{2}(t+\varepsilon )dt-\underset{\text{Autocorrelation}}{\underset{︸}{\frac{1}{2}\int_{-\infty }^{+\infty }y_{1}(t)y_{1}(t+\varepsilon )dt}} \end{array}$$

A signal of interest (such as cavitation) *η*(*t*) is then added to the heart signal so that $y_{2}(t)=y_{1}(t+\varepsilon )+\underset{\text{Cav/noise}}{\underset{︸}{\eta (t)}}$. Thus equation (16) becomes

(17) $$\begin{array}{l} E_{non-\det}=\overset{\text{same as previous equation}}{\overset{︷}{\frac{1}{4}\int_{-\infty }^{+\infty }y_{1}^{2}(t)dt}}\\ +\overset{\text{same as previous equation}}{\overset{︷}{\frac{1}{4}\int_{-\infty }^{+\infty }y_{1}^{2}(t+\varepsilon )dt-\frac{1}{2}\int_{-\infty }^{+\infty }y_{1}(t)y_{1}(t+\varepsilon )dt}}\\ +\frac{1}{4}\left(2\right)\int_{-\infty }^{+\infty }y_{1}(t+\varepsilon )\eta (t)dt\\ -\frac{1}{2}\int_{-\infty }^{+\infty }y_{1}(t)\eta (t)dt+\underset{\text{Cavitation/noise}}{\underset{︸}{\frac{1}{4}\int_{-\infty }^{+\infty }\eta^{2}(t)dt}} \end{array}$$

From the non-deterministic energy result in (17), one can observe that if *ε *= 0, then

(18) $$E_{non-\det}=\frac{1}{4}\int_{-\infty }^{+\infty }\eta^{2}(t)dt$$

which implies that the non-deterministic energy represents only the non-repeating portions of the signal, as expected and previously shown. However, if *ε *≠ 0, then *ε *appears in the non-deterministic energy result as shown in (17), meaning that the non-deterministic energy is *not *a measure of *η*(*t*)alone. Therefore, if the beats are not properly superimposed, those beats that are not lined up will have an impact on the non-deterministic result, which might be falsely interpreted as a greater level of the non-repeating portion in the signal.

#### 2.2.3. Calculations with a test sine signal

To calculate the relative sizes of contributions to the energies from the heartbeat portion compared with the non-deterministic portion, test sine signals were used since they are periodic and allow for simple calculations in closed form. A test signal was constructed consisting of a simple low-frequency sine signal, which represents the heartbeat, along with a higher frequency sine signal representing an extremely simple cavitation signal.

(19) $$\begin{array}{l} y_{1}(t)=\underset{\text{Heart beat}}{\underset{︸}{A_{1}\sin(\Omega t)}}+\underset{\text{Cavitation}}{\underset{︸}{C_{1}\sin(\omega_{1}t+\phi_{1})}}\\ y_{2}(t)=\underset{\text{Heart beat}}{\underset{︸}{A_{2}\sin(\Omega t)}}+\underset{\text{Cavitation}}{\underset{︸}{C_{2}\sin(\omega_{2}t+\phi_{2})}} \end{array}$$

Here, *A*_*i *_is the amplitude of the *i*th heart beat signal, *C*_*i *_is the amplitude of the *i*th cavitation signal, Ω is the frequency of the heart signal, *ω*_1 _and *ω*_2 _are the (higher) frequencies of the cavitation signal, and *φ*_*i *_is the phase shift for each cavitation signal. A phase shift is required to ensure the cavitation signal will not be in-phase with the heartbeat. Using the parameters, T=2πΩ, *ω*_1 _= 35000Ω Hz, *ω*_2 _= 36000Ω Hz so that the cavitation has a much higher frequency than the heartbeat, and also *A*_1 _= *A*_2 _= *A*, *C*_1 _= *C*_2 _= *C*, *φ*_1 _= *φ*_2 _= 0, *y*_1_(*t*) ≠ *y*2(*t*), the deterministic energy is found to be

(20) $$E_{\det}=\frac{1}{2}\frac{\pi \left(C^{2}+2A^{2}\right)}{\Omega }$$

The value of *ω*_1 _was chosen as 35 kHz times the value of Ω since the high frequency pressure fluctuations due to cavitation occur in the frequency range of 35 kHz to 350 kHz [39]. The frequency *ω*_2 _was assigned a different value than *ω*_1 _to account for possible variations in frequency between heart beats. The value of Ω is approximately 1 Hz since the duration of a heart beat is approximately 1 second. If the value of *ω*_1 _and *ω*_2 _is changed to any integer between the frequency range mentioned previously, the deterministic energy equation in (20) will remain the same. From equation (20), it is noted that the amplitudes squared of both the heart-beat and cavitation signals appear, with the heart-beat itself having a stronger presence due to the factor 2.For the chosen signals, the total energy can be shown to be given by

(21) $$E_{total}\;=\frac{\pi \left(A^{2}+C^{2}\right)}{\Omega }$$

The non-deterministic energy is obtained by subtracting the deterministic energy from the total energy:

(22) $$\begin{array}{l} E_{non-\det}=\frac{\pi \left(A^{2}+C^{2}\right)}{\Omega }-\frac{1}{2}\frac{\pi \left(C^{2}+2A^{2}\right)}{\Omega }\\ \;\;\;\;\;\;\;\;\;\;=\frac{1}{2}\frac{\pi C^{2}}{\Omega } \end{array}$$

It is observed in (22) that *E*_non-det _does *not *depend on *A*, the amplitude of the heart signal, although both the deterministic and total energies do. It depends on C, the amplitude of the cavitation signal. Therefore, for perfectly aligned heart beats, the non-deterministic energy represents the cavitation in the signal confirming the algorithm.

#### 2.2.4. Superposition problem

When heart beats are not perfectly superimposed, there are two different possibilities after truncation:

The first possibility is that *y*_2_(*t*), the second heart beat, will be zero for the first few points since it is slightly shifted to the right due to its misalignment. This will resemble a misplaced heart beat since the value before the first heart sound in a heartbeat should be near zero. The second possibility is that the beginning of *y*_2_(*t*) will be the part of the beat that was cut off at the end by the truncation, since it is slightly shifted to the right. Some calculations are demonstrated below to compare the results obtained with the two possibilities mentioned previously. The calculations are done using two heart beats.

##### 1^st ^case

The test signal used is identical to the one used in (19).

Now, the second heart beat is "misaligned" so that *y*_2_(*t*) = *y*_1_(*t *- *ε*)*u*(*t *- *ε*), where *u*(*t *- *ε*)is the unit step function and the heart beats are truncated to have length *T.*

With the chosen form of the test signal in (19), and the parameters *ω*_1 _= 35000 Hz, Ω = 1, *φ*_1 _= *φ*_2 _= 0, *ε *= 10*ms*, this gives

(23) $$\begin{array}{l} E_{non-\det}=\frac{1}{4}A^{2}\pi +\frac{1}{4}C^{2}\pi \\ \;\;\;\;\;\;\;\;\;\;=0.7854A^{2}+0.7854C^{2} \end{array}$$

Note that with misalignment of the beats explicitly modelled as a shift in one of the heart beats, the non-deterministic energy now depends on the magnitude of the cavitation signal *as well as *the magnitude of the heart beats themselves and the dependence on both is about the same, with both factors having a π/4 dependence. When the signals were perfectly aligned, then using the same parameters, equation (22) indicates that the nondeterministic energy was π2C2 whereas the nondeterministic energy with misaligned beats is π4A2+π4C2. The contribution of the cavitation portion of the signal has effectively halved and the heart-beat portion now has an equally weighted contribution. This is potentially particularly problematic as in general the heart beat is louder than any 'signal of interest', implying that *A *will be much larger than *C *in the previous equation. Thus, in this case the level of non-deterministic energy will rise - and *not *due to an increase in non-heartbeat signals but due to a misalignment of the beats.

##### 2^nd ^case

If we assume that *y*_2_(*t*) = *y*_1_(*t-ε*), then using the same form of signals and same parameters as the previous calculation, the non-deterministic energy becomes

(24) $$E_{non-\det}=2.8888A^{2}+1.3747C^{2}$$

Once again, with the effect of the misalignment of the beats accounted for, it can be seen that the non-deterministic energy depends on the magnitudes of *both *the cavitation and heart beat signals. The result obtained with the first possibility in (23) seems better than the result obtained with the second possibility in (24) since *A*, the amplitude of the heart beat signal, contributes less to the non-deterministic energy (0.7854*A*^2 ^vs. 2.8888*A*^2^). In equation (23), the weighting of the contribution of A^2 ^and C^2 ^to the nondeterministic energy is equal whereas in equation (24), the contribution of A^2 ^is about twice as much as the contribution of C^2^. Ideally, the component coming from the heart beat should not be present in the non-deterministic energy since the latter is supposed to represent the non-heartbeat portion of the signal only.

Since the quality of the segmentation of the heart signal has a large impact on how well the heart beats are superimposed and thus the calculation of energies of interest, we propose to implement an algorithm that aligns the heart beats in the signal after the initial segmentation has been performed.

**Figure 3:**
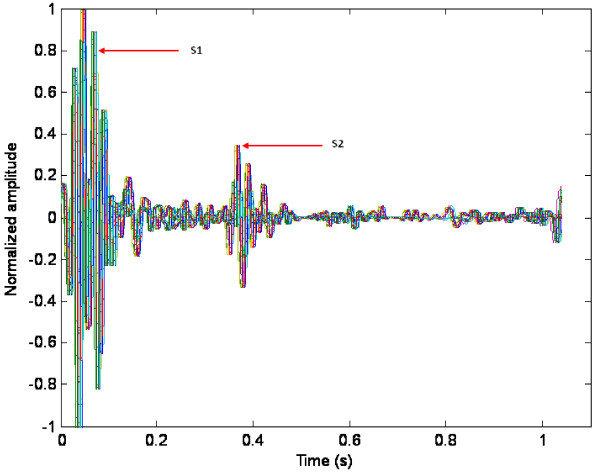
**Superimposed and truncated heart beats of the pre-recorded stethoscope test signal**.

To demonstrate the need for further beat alignment, even after the initial segmentation is performed; Figure 3 shows the superimposed heart beats of the pre-recorded stethoscope test signal (Pre-recorded PCG 1) after the initial segmentation was performed. The first large peak of activity in the heart beats of Figure 3 is the first heart sounds (S1) and the second peak of activity is the second heart sounds (S2). The heart beats in Figure 3 are not perfectly lined up, which is not surprising since perfect alignment is practically impossible as the heart does not produce a perfectly periodic heart signal. Therefore, the signals were processed so as to align all the S1's in the heart signal after all the heart beats have been superimposed. To do this, the maximum peak of the first quarter of each heart beat, which is the peak of S1, was determined. The S1 is usually contained in the first quarter of the heart beat. The algorithm searches for the maximum peak in the first quarter of the heart beat instead of in the entire heart beat since the S2 peak can sometimes be higher than the S1 peak, in which case Matlab would detect the S2 peak as the maximum peak instead of S1. After the S1 peaks were found for each individual heart beat, each heart beat was shifted to the left or to the right to line up with the S1 of the first heart beat of the heart signal. Once all the S1's were lined up, the beginning or the end of the heart beats were truncated so that all the beats have the same length. The larger the shift, the more the heart beat is going to be truncated. The beats that require a very large shift compared to the other beats may eventually removed from the heart signal since it is considered a 'bad' heart beat. Too much of that heart beat would be truncated after shifting; hence, too much information would be lost. The more signals are lined up, the higher the ensemble average will be.

## 3. Results and Discussion

### 3.1. Results of Aligning the S1 peaks in the signal

The acquired signals were normalized at the beginning of the algorithm to allow for the comparison of signals from different patients if needed. Some patients have quieter heart beats than others. Hence, the figures and calculated energy results are dimensionless. Furthermore, all analyzed signals consisted of 18 heart beats. To demonstrate the impact of peak alignment after initial segmentation, the energies were calculated with and without the S1's aligned; Table 1 shows the energy results and the percentage ratio of non-deterministic energy to total energy in the signal.

**Table 1:** Comparison of the results obtained with and without the S1's aligned

Signal used	S1	% of non-det energy	Energy		
			
			Deterministic energy	Total energy	Non-deterministic energy
**Pre-recorded PCG 1**	**Not aligned**	73.20	266.42	994.00	727.59
	
	**Aligned**	35.18	624.23	962.98	338.76

**Pre-recorded PCG 2**	**Not aligned**	88.17	106.21	898.09	791.88
	
	**Aligned**	6.82	836.76	897.99	61.23

**Pre-recorded PCG 3**	**Not aligned**	71.59	432.08	1.52*10^3^	1.09*10^3^
	
	**Aligned**	47.81	781.33	1.50*10^3^	715.65

**Karim stethoscope signal 1**	**Not aligned**	51.29	229.67	471.52	241.85
	
	**Aligned**	48.40	225.76	437.49	211.73

**MHV Recording 1**	**Not aligned**	95.74	25.31	593.73	568.42
	
	**Aligned**	77.81	130.89	589.89	458.99

The sampling frequency used for the stethoscope signals and for the hydrophone signal was 44.1 kHz.

Comparing the results obtained with and without the S1's aligned, one can observe in Table 1 that for the first four signals, the results improved with the S1's aligned. The percentage was lower with the S1's aligned, which is a better representation of the absence of cavitation. The misplaced heart beats were causing the large difference in percentages between the signals with and without the S1's aligned. The algorithm is thus very sensitive to the lining up issues and greatly depends on the quality of the segmentation algorithm. The results were also improved with the S1's aligned in the fifth signal, which was recorded with a mechanical heart valve using a left-heart simulator at the University of Ottawa Cardiovascular Mechanics Lab. However, hydrophone signals are very noisy; therefore the percentage remains high. Additionally, since that signal was a hydrophone recording of the mechanical heart valve, it could possibly have a cavitation component in it which could possibly be another explanation for the higher percentage, although no cavitation was visually detected during the recording.

### 3.2. Test for the quality of the segmentation

This additional processing to the algorithm can also be used as a test of the quality of the initial segmentation by looking at the S1 shift values. Whether the quality of the input heart signal was bad or the segmentation was poorly done, the algorithm helps the user to choose whether to discard the signal if the majority of the S1 shifts are too large, or to simply remove the beats that require a large S1 shift. If 'bad' heartbeats are to be removed, then the user would need to establish a priori the threshold shift for determining what level of required shift is considered unacceptable. The steps taken for our analysis will first be described in detail below for the first signal (Pre-recorded PCG 1).

The shifts required to align all the S1 peaks with the S1 peak of the first heart beat of the heart signal are shown in Table 2. The shift number represents the number of samples by which the beat needed to be shifted. A negative number represents a left shift and a positive number represents a right shift. There are 18 beats in this stethoscope signal (Pre-recorded PCG 1).

**Table 2:** Shifts for the signal Pre-recorded PCG 1

Heart beat number	Shift in samples
1	0

2	-679

3	102

4	-1018

5	33

6	-1087

7	135

8	-985

9	271

10	-849

11	-68

12	-747

13	304

14	-816

15	-35

16	-714

17	61

18	-1059

In this signal, there are a total of 44,474 samples (1.0085 sec) in each heart beat after truncation. The average shift in samples is 497.94 (11.29 msec) and the standard deviation in the shifts in samples is 417.78 (9.47 msec). The cutoff for the Pre-recorded PCG 1 signal was chosen to be a shift of magnitude 1000 (22.67 msec) as the majority of heart beats had shifts of less than 1000 samples. Any beat with a shift larger than 1000 will be removed prior to calculation of any energies. Thus, beats number 4, 6 and 18 were removed in this signal. Table 3 shows the energy results and the percentage of non-deterministic energy in the stethoscope signal with all the beats, and with the three beats removed.

**Table 3:** Energy results for the Pre-recorded PCG 1 signal with all beats and with three beats removed

Signal used	Beats	% of non-det energy	Energy		
			
			Deterministic energy	Total energy	Non-deterministic energy
**Pre-recorded PCG 1**	**All beats**	35.18	624.23	962.98	338.76
	
	**Beats 4, 6 and 18 removed**	33.11	656.68	981.71	325.03

Now, the average shift in samples is 386.60 and the standard deviation in the shifts in samples is 363.41. Comparing the results obtained with all beats and with three beats removed, one can observe that the results were slightly improved with removed beats due to improved lining up of the beats. The lower percentage is a better representation of the absence of cavitation.

The same steps were implemented with the four remaining signals and the results are summarized in Table 4. It includes the energy results and the percentage of non-deterministic energy for four different signals with all the beats included, and with some beats removed. The cutoff for the signal Pre-recorded PCG 2 was chosen to be a shift of 250 as the majority of heart beats had shifts of less than 250. The shift values for this signal were much smaller than the one for the signal Pre-recorded PCG 1. The cutoff for the signal Pre-recorded PCG 3 was chosen to be a shift of 770, and for the signal Karim stethoscope signal 1, it was chosen to be 500. Finally, the cutoff for the signal MHV Recording 1 was chosen to be a shift of 850. The cutoff values for each signal were chosen by evaluating the shift values; the ones that were large compared to the others were removed, which explains why every signal has a different number of beats removed.

**Table 4:** Results obtained with all beats and with some beats removed

Signal used (samples in HB)	Beats	Ave shift (sam-ples)	STD shift (sam-ples)	% of non-det energy	Energy		
					
					Det energy	Total energy	Non-det energy
**Pre-recorded PCG 2 (HB: 46671)**	**All beats**	127.06	86.98	6.82	836.76	897.99	61.23
	
	**Beats 2 and 6 removed**	107.38	68.96	6.87	816.82	877.10	60.27

**Pre-recorded PCG 3 (HB: 36672)**	**All beats**	470.27	304.46	47.81	781.33	1.50*10^3^	715.65
	
	**Beats 17, 20 and 21 removed**	419.32	296.65	44.60	843.53	1.52*10^3^	678.99

**Karim stethoscope signal 1 (HB: 34861)**	**All beats**	117.52	280.32	48.40	225.76	437.49	211.73
	
	**Beats 5 and 18 removed**	36.89	70.89	41.86	273.14	469.80	196.66

**MHV Recording 1 (HB: 36510)**	**All beats**	499	296.24	77.81	130.89	589.89	458.99
	
	**Beats 8, 11, 21, 22 removed**	406.28	240.36	73.56	156.91	593.44	436.53

In Table 4, HB is heart-beat, Ave is average, STD is standard deviation, det is deterministic and non-det is non-deterministic. Comparing the results obtained in Table 4, one can observe that the results were slightly improved with removed beats for all signals with the exception of the Pre-recorded PCG 2 signal. However, the Pre-recorded PCG 2 signal already has a very low percentage of non-deterministic energy and its shift values are very small, which is why removing some beats barely made a difference in the results. It was already a good representation of the absence of cavitation.

Thus, we conclude that by removing 'bad' heartbeats from the signal prior to computing the energies, improvement in the results can be expected. However, the level at which the shift of a heartbeat requires its removal from consideration has not been established and should be considered carefully before being implemented.

### 3.3. Aligning the S2 peaks in the signal

Next, we investigated the impact of aligning the S2 peaks instead of the S1 peaks, after all the heart beats have been superimposed. To do this, the maximum peak of the last three-quarters of each heart beat was determined. The S2 peak is generally contained in the last three-quarters of the heart beat. After the S2 peaks were found, they were all shifted to the left or to the right to line them up with the S2 peak of the first heart beat of the heart signal. Then, the beginning or the end of the heart beats was truncated for all beats to have the same length. The larger the shift, the more the heart beat is going to be truncated. As mentioned in the previous sub-section, the beats that require a very large shift compared to the other beats are removed from the heart signal since it is considered a 'bad' heart beat. Too much of that heart beat would be truncated after shifting; hence, too much information would be lost.

To demonstrate the impact, the simulations were done with and without the S2's aligned; Table 5 showing the energy results and the percentage of non-deterministic energy in the signal.

**Table 5:** Comparison of the energy results obtained with and without the S2's aligned

Signal used	S2	% of non-det energy	Energy		
			
			Deterministic energy	Total energy	Non-deterministic energy
**Pre-recorded PCG 1**	**Not aligned**	73.20	266.42	994.00	727.59
	
	**Aligned**	29.87	691.23	985.57	294.35

**Pre-recorded PCG 2**	**Not aligned**	88.17	106.21	898.09	791.88
	
	**Aligned**	6.16	842.66	897.96	55.29

**Pre-recorded PCG 3**	**Not aligned**	71.59	432.08	1.52*10^3^	1.09*10^3^
	
	**Aligned**	63.47	544.78	1.49*10^3^	946.43

**Karim stethoscope signal 1**	**Not aligned**	51.29	229.67	471.52	241.85
	
	**Aligned**	77.49	96.72	429.71	332.98

**MHV Recording 1**	**Not aligned**	95.74	25.31	593.73	568.42
	
	**Aligned**	93.21	32.05	472.10	440.05

Comparing the results obtained with and without the S2's aligned, one can observe that for the majority of the signals, the results were improved with the S2's aligned meaning that the percentage of non-deterministic energy was reduced. The only exception is the signal 'Karim stethoscope signal 1', where the results were worse than before the alignment. The percentage results obtained with the S1 peaks aligned and with the S2 peaks aligned are summarized in Table 6.

**Table 6:** Comparison of the percentage results obtained with the S1 peaks and with the S2 peaks aligned

Signal used	% of non-deterministic energy	
	
	S1 peaks aligned	S2 peaks aligned
**Pre-recorded PCG 1**	35.18%	29.87%

**Pre-recorded PCG 2**	6.82%	6.16%

**Pre-recorded PCG 3**	47.81%	63.47%

**Karim stethoscope signal 1**	48.40%	77.49%

**MHV Recording 1**	77.81%	93.21%

The percentage of non-deterministic energy for the two first signals was slightly better with the S2 peaks aligned rather than with the S1 peaks aligned. However, the percentage was much better for the last three signals with the S1 peaks aligned. Additionally, for the signal 'Karim stethoscope signal 1', the percentage result was slightly better with the S1 peaks aligned compared to the results with no peaks aligned, but was worst with the S2 peaks aligned.

When using the improved algorithm, it is recommended that the non-deterministic energy should be calculated with the S1 peaks aligned and with the S2 peaks aligned. Then, the alignment method giving the best result between the two is the one that should be used in the final calculation. However, for the purpose of the rest of this article, the S1 peak alignment method was used in the algorithm and no beats were removed.

### 3.4. Time-frequency Analysis

We have shown so far that a calculation of the non-deterministic energy in a signal can be an effective way to separate repeating (heart-beat) like components of a heart signal from other signals of interest, as long as the segmentation of the signal is done carefully. This gives a measure of energy content per heartbeat but temporal occurrence of a specific event is lost. Consequently, the energy cannot be localized to a certain time. It would be useful to have a time-frequency method that enables us to analyze and interpret the time-varying energy contents [40]. It would be interesting to know when the high frequencies occur in the heart signal, for example since cavitation bubble implosion creates high-frequency pressure fluctuations when it is present. In fact, the preceding analysis can be easily extended via the Short-Time Fourier Transform (STFT) to yield time-localized information about the energy content of each heart-beat.

First, a non-stationary signal *x*(*t*) is segmented into quasi-stationary parts *x*_*k*_(*n*) by applying a moving window to the signal. Then, the Fourier Transform is computed for the *k*^th ^segment as

(25) $$X_{k}(\omega )=\sum_{n=0}^{M-1}x_{k}(n)\cdot e^{-j\omega n}$$

The array of spectra *X*_*k*_(*ω*)for *k *= 1, 2,..., *K *will describe the time-varying spectral characteristics of the signal [41]. The *k*^th ^segment *x*_*k*_(*n*) may be expressed as the multiplication of the signal *x*(*n*) with a window function *w*(*n*) which may be positioned at any time instant *m*. The resulting segment may be expressed as *x*(*n*)*w*(*n *- *m*). In practice, the Fourier Transform should not have to be computed for every possible window position, that is, for every *m*. To reduce the computation time, it is common practice for the adjacent windows to overlap for half of the segment samples. Overlapping is desirable in order to maintain continuity in the STFT [41]. The Fourier Transform of every segment becomes

(26) $$X(m,\omega )=\sum_{n=0}^{M-1}x(n)w(n-m)\cdot e^{-j\omega n}$$

The expression given in (26) is the Short-Time Fourier Transform. The STFT modulus square |*X*(*m*,*ω*)|^2^is called a spectrogram [40,42]. In Matlab, the spectrogram can be displayed in a 2D or 3D representation.

The limitation imposed by the use of a window is related to the uncertainty principle, which states

(27) $$\Delta t\times \Delta \omega \geq \frac{1}{2}$$

where Δ*t *is the time extent (duration) of the signal *x*(*t*) and Δ*ω *is the frequency extent (bandwidth) of its Fourier Transform *X*(*ω*). The limitation implies that it is not possible to simultaneously obtain a high time and frequency resolution [41].

### 3.5. Implementation of the time-frequency analysis

There are a few advantages to using the STFT instead of the Fourier Transform in the algorithm. First, with the STFT, it is possible to know when the high frequencies occur in the heart beats by looking at the 2D and 3D spectrogram representations of the total heart signal. Additionally, it is possible to plot the non-deterministic energy values against time with the STFT, thus allowing visualization of the location of the non-deterministic energy in the heart beat. Finally, similar steps as used previously were followed to find the non-deterministic energy with the STFT, and it provided more information about the location of energy in the signal without complicating the algorithm.

The STFT of the signals was computed in Matlab using the function *spectrogram *included in the Signal Processing Toolbox. The spectrogram is the magnitude of the STFT. In the algorithm, the window was chosen to be a Hamming window of the same length as the FFT. The number of samples by which the consecutive segments overlap was chosen to be half the length of the FFT, which produces 50% overlap between segments. It was chosen by trial and error to be 1024 since it gave a good time resolution and an acceptable frequency resolution. In order to provide a reasonably good estimate of when the high frequency components such as cavitation occur in the heart beats, a better time resolution is preferred. The sampling frequency used was Fs = 44.1 kHz.

The deterministic energy and the total energy can be calculated in the frequency domain using the STFT. The energy density spectrum of the STFT of the ensemble average was calculated, from which the deterministic energy was found. In this manner, the result obtained is a time evolution of the deterministic energy in the signal. The total energy was similarly found via the spectrogram followed by the energy density spectrum of the STFT of each heart beat in the signal. The result obtained is a time evolution of the total energy in the signal.

**Figure 4:**
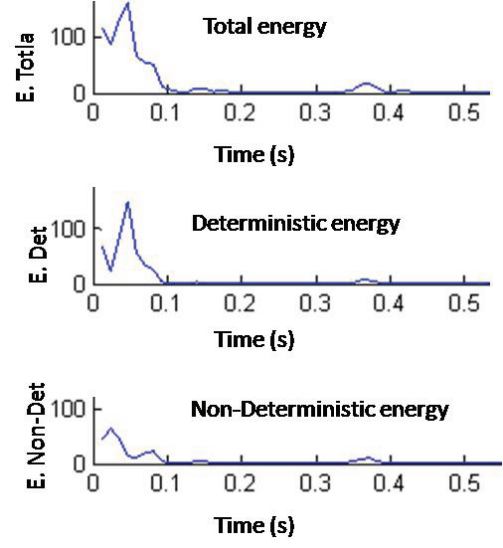
**The time evolution of the total, deterministic, and non-deterministic energy**.

The non-deterministic energy is found by subtracting the deterministic energy from the total energy giving a time evolution of the non-deterministic energy. The deterministic energy, the total energy and the non-deterministic energy are all demonstrated in Figure 4. This extension to the original algorithm allows us to see the non-deterministic values as they evolve across a heart-beat. The first and second lumps in the non-deterministic energy plot most likely represent the parts of the S1 and S2 peaks that were not perfectly lined up. The rest of the non-deterministic energy most likely comes from the noise in the signal.

In order to know if the results using the STFT agree with the results using the Fourier Transform, the percentage of non-deterministic energy obtained with both methods are compared in Table 7 for a few signals. To obtain the total energy of the whole signal as represented by a single number, the total energy over time is summed. The same is done for the non-deterministic energy. Note that for the all signals in both methods, the S1 peaks were aligned and no heart beats were removed from the signal.

**Table 7:** Comparison of the percentage results obtained with the STFT and with the FT

Signal used	% of non-deterministic energy	
	
	Fourier Transform	Short-Time Fourier Transform
**Pre-recorded PCG 1**	35.18%	36.11%

**Pre-recorded PCG 2**	6.82%	6.87%

**Pre-recorded PCG 3**	47.81%	46.81%
**Karim stethoscope signal 1**	48.40%	45.17%

As observed in Table 7, the percentage results obtained with both methods are very similar. Therefore, the Short-Time Fourier Transform can be used instead of the Fourier Transform to obtain the energies in the signal, with the added advantage that the time-evolution of energy in a heart-beat can be displayed if desired.

## 4. Conclusions

The algorithm presented in this paper was based on ideas proposed by Johansen for the detection of cavitation in mechanical heart valve patients. Johansen's algorithm was chosen for further analysis as it presented the most potentially effective algorithm presented in the literature to date that had actually been implemented on biological signals acquired in vivo. A closed-form mathematical analysis of the algorithm was presented and demonstrated analytically that the algorithm will quantify levels of non-deterministic energies, as long as proper signal segmentation is performed so that all heart beats are superimposed as much as possible. It can be concluded that the theory behind the algorithm presented in this paper is sound, and that the non-deterministic energy can indeed represent the non-repeating components in the signal if the heart beats are perfectly aligned and if there is no random noise in the signal. However, any error in perfectly lining up the repeating heartbeats will lead to another additional contribution to the non-deterministic energy which could lead to a false interpretation of the presence of an additional quantity of 'signal of interest'. To improve the algorithm, it was proposed to aligning the S1 and S2 peaks in the signal subsequent to the initial segmentation. As a result, it reduced the amount of misplaced heartbeats and the non-deterministic energy became a better representation of the 'signal of interest'. If a time evolution of the energies in the signal is desired then the Short-Time Fourier Transform permits the observation of the non-deterministic energy values as they evolve across a heartbeat and it makes it possible to see where the non-deterministic energy is located in the signal. This additional information is provided while preserving the same energy result accuracy of the algorithm that uses the Fourier Transform alone.

In this paper, we have endeavored to carefully analyze the theory behind the algorithm and to work with real signals acquired in vivo in order to investigate the types of difficulties that can arise with the use of actual signals. It is our belief that this energy approach to heart signal analysis is simple yet powerful enough to provide meaningful results when properly used. For a proper application of these results, a careful and methodical signal acquisition protocol must be designed and implemented by the end user. In particular, end users also need to determine appropriate quality of signals to choose to process as signals that are excessively noisy will lead to poor results and should be rejected from further signal processing considerations. The levels that are considered to be excessively noisy will depend on the application and equipment used for signal acquisition and should be appropriately determined based on these factors, rather than on general recommendations. Similarly, we have not attempted to determine appropriate levels of non-deterministic energies since again, these will and should depend on the application, quantity of interest and equipment used. What we have presented is a signal-processing tool that, with care, can be used in a larger scale study in order to determine those levels of signal-of-interest that are considered appropriate, too noisy or a sign of pathology.

## Related Website

Additionally, for the interested reader, we have created a website where some of the signals used, Matlab code written, conference papers and master's thesis related to this work can all be found. This is located at http://sites.google.com/a/uottawa.ca/signal-processing-of-heart-signals/home.

## Competing interests

The authors declare that they have no competing interests.

## Authors' contributions

VM carried out the data acquisition and wrote the code for the implementation of the algorithms. NB suggested and oversaw the mathematical analysis and algorithms. Both authors contributed to the interpretation of results and to the writing of the article. Both authors have read and approved the final manuscript.

## Authors' information

VM recently completed her master's degree in Biomedical Engineering at the University of Ottawa and the subject of this article was the focus of her master's thesis.

NB holds a PhD from the University of Toronto and is currently an associate professor at the University of Ottawa in the Department of Mechanical Engineering.
